# Epigallocatechin gallate reverses cTnI‐low expression‐induced age‐related heart diastolic dysfunction through histone acetylation modification

**DOI:** 10.1111/jcmm.13169

**Published:** 2017-04-06

**Authors:** Bo Pan, Junjun Quan, Lingjuan Liu, Zhongwei Xu, Jing Zhu, Xupei Huang, Jie Tian

**Affiliations:** ^1^ Heart Centre Children's Hospital of Chongqing Medical University Chongqing China; ^2^ Key Laboratory of Developmental Disease in Childhood (Chongqing Medical University) Ministry of Education Chongqing China; ^3^ Key Laboratory of Pediatrics in Chongqing Chongqing China; ^4^ Chongqing International Science and Technology Cooperation Center for Child Development and Disorders Chongqing China; ^5^ Department of Biomedical Science Charlie E. Schmidt College of Medicine Florida Atlantic University Boca Raton FL USA

**Keywords:** histone acetylation, age‐related cardiac diastolic dysfunction, cardiac troponin I, epigallocatechin‐3‐gallate, HDACs

## Abstract

Cardiac diastolic dysfunction (CDD) is the most common form of cardiovascular disorders, especially in elderly people. Cardiac troponin I (cTnI) plays a critical role in the regulation of cardiac function, especially diastolic function. Our previous studies showed that cTnI‐low expression induced by histone acetylation modification might be one of the causes that result in diastolic dysfunction in ageing hearts. This study was designed to investigate whether epigallocatechin‐3‐gallate (EGCG) would modify histone acetylation events to regulate cTnI expression and then improve cardiac functions in ageing mice. Our study shows that EGCG improved cardiac diastolic function of aged mice after 8‐week treatment. Low expression of cTnI in the ageing hearts was reversed through EGCG treatment. EGCG inhibited the expression of histone deacetylase 1 (HDAC1) and HDAC3, and the binding levels of HDAC1 in the proximal promoter of cTnI. Acetylated lysine 9 on histone H3 (AcH3K9) levels of cTnI's promoter were increased through EGCG treatment. Additionally, EGCG resulted in an ascent of the binding levels of transcription factors GATA4 and Mef2c with cTnI's promoter. Together, our data indicate that EGCG may improve cardiac diastolic function of ageing mice through up‐regulating cTnI by histone acetylation modification. These findings provide new insights into histone acetylation mechanisms of EGCG treatment that may contribute to the prevention of CDD in ageing populations.

## Introduction

Age‐related changes in cardiovascular structure/function are implicated in the markedly increased risk for cardiovascular disease in older persons 1, 2. Plenty of studies have revealed that diastolic dysfunction increases with advancing age; nearly 40% of the population (aged 45 or older) had diastolic dysfunction 3, 4, 5. CDD was predictive of all‐cause mortality, even when controlling for sex, age and ejection fraction, and can also be assumed as an independent predictor of failure events 3. However, there are still lacks of effective interventions targeting on CDD.

cTnI, an inhibitory subunit of troponin complex, can bind to actin–tropomyosin and prevent muscle contraction through inhibiting the actin‐activated myosin (actomyosin) ATPase activity, and regulates cardiac systolic/diastolic function dynamically 6. cTnI degradation has been reported in myocardial cells after ischaemia and cardiac stunning. cTnI may significantly decrease in post‐infarction LV remodelled myocardium remote from the infarct zone, and a study using a small sample of human heart tissues showed that the content of cTnI in LV myocardium may decrease in older men with or without cardiac disease. Our previous studies have demonstrated that progressive troponin I loss impairs cardiac relaxation and causes heart failure in mice 7, and our most recently published data showed that hypoacetylation of AcH3K9 near the key cis‐elements of cTnI's proximal promoter might cause a cTnI decrease in ageing hearts 8. However, the underlying mechanisms are still less obvious.

Epigallocatechin‐3‐gallate (EGCG), a major polyphenol in green tea, has been extensively studied as a bioactive dietary component against various types of carcinomas through multiple mechanisms such as antioxidation, induction of apoptosis, inhibition of angiogenesis and metastasis 9. In recent studies, EGCG has been studied for a considerable role in epigenetic regulation, such as inhibiting DNA methyltransferase and reactivating methylation‐silenced genes, and having the ability to inhibit class I HDACs (HDAC1, HDAC2 and HDAC3)^10^, ^11^, ^12^.

In this study, we found an increase in HDAC1 in ageing hearts and a higher binding level of HDAC1 with cTnI gene's promoter, which might result in a hypoacetylation of histone 3 lysine 9, and that chromatin regulation caused lower binding levels of transcription factors with cTnI's promoter. Further investigations showed that EGCG could inhibit the expression of HDAC1 and its binding levels with cTnI's proximal promoter and then reverse cTnI‐low expression and improve cardiac diastolic function.

## Materials and methods

### Animals and treatment

Healthy and 3‐month‐, 16‐month‐ and 18‐month‐old SPF class c57bl/6 mice were purchased from the Experimental Animal Center in Chongqing Medical University (Chongqing, China). All procedures on experimental animals were approved by the Animal Care and Use Committee at the Chongqing Medical University. Animal experiments were performed in accordance with the NIH guidelines (Guide for the care and use of laboratory animals)^13^. Sixteen‐month‐old mice were treated with single dose (50 mg/kg/day) of EGCG (Selleck) and dimethyl sulphoxide (DMSO, dissolvent control) by intraperitoneal injection for 8 weeks. The mice were killed by carbon dioxide asphyxia, and cardiac tissues were collected.

### Morphological observations

Cardiac ultrastructure was observed through transmission electron microscope. Heart tissue collection and fixation was performed as previously described. Generally, cardiac tissue of left ventricle was cut into small pieces, about 1 mm^3^, and fixed immediately in 2.5% glutaraldehyde for about 12 hrs. Fixed heart samples were sent to TEM centre of Chongqing Medical University, and samples were sliced by ultramicrotome (Leica, Solms, Germany) and stained negative using lead citrate. Sample observation was performed, and images were taken using a transmission electron microscope (Hitachi, Tokyo, Japan).

### Terminal deoxynucleotidyl transferase dUTP nick‐end labelling (TUNEL) assay

A terminal deoxynucleotidyl transferase‐mediated dUTP nick‐end labelling (TUNEL) assay was performed according to the manufacturer's instructions (*In Situ* Cell Detection Kit; Roche, Mannheim, Germany). The nuclei were counterstained with DAPI in blue. TUNEL‐positive cells were marked in green under a fluorescent microscope (Nikon, Tokyo, Japan).

### Echocardiography

Echocardiographic studies were performed using Panoview‐β1500 (Panoview Taiwan). To decrease experimental bias, all of the echocardiography measurements were performed by an examiner blinded to the animals' age. Experimental mice were anaesthetized with pentobarbital sodium at a concentration of 1% by intraperitoneal injection, 7 μl/g. Hair on the precordial region was cleanly removed with VEET hair remover (Reckitt Benckiser, London, UK), and region was covered with ultrasound transmission gel (An Xinchao Chongqing, China). Left ventricle short‐axis images were taken to view the LV movement during diastole and systole, allowing us to measure the ventricle structure and dimension. Transmitral blood flow was observed Pulse Doppler. All data images were saved and analysed with used an automated analysis or semi‐automated analysis software (Panoview Taiwan) to evaluate the cardiac function.

### Western blotting

Western blotting assays for cTnI and acetylated histone 3 (acH3) were performed as previously described 8, 14. An anti‐cTnI monoclonal antibody (cTnI) that recognized mouse cTnI was used at a dilution of 1:1000 (Abcam; Cambridge, UK, ab47003), anti‐acH3 (MILLIPORE 17‐615) and total histone 3 (H3) monoclonal antibodies (ab1791) were used at a dilution of 1:2000, and anti‐β‐actin monoclonal antibody (BOSTER, Wuhan, China) was used at a dilution of 1:1000. The immune‐reactive protein bands were visualized with Chemiluminescence Luminol Reagent (Merck Millipore, USA). After scanning, protein bands were analysed with Quantity One version 4.4 software (Bio‐Rad, CA, USA).

### Total RNA extraction and quantitative real‐time PCR analysis

The real‐time PCR was carried out as described previously 8, 14. β‐Actin was used as the endogenous ‘housekeeping’ gene to normalize the RNA sample levels. The primer sequences of cardiac‐specific genes and controls were designed as follows: cTnI: 5′‐GCAGGTGAAGAAGGAGGACA‐3′ (forward) and 5′‐CGATATTCTTGCGCCAGTC‐3′ (reverse); β‐actin: 5′‐CACACCCGCCACCAGTTCG‐3′ (forward) and 5′‐GTCCTTCTGACCCATTCCCACC‐3′ (reverse); HDAC1: 5‐ATGAGCTGCCCTACAACGAC‐3′ (forward) and 5′‐GACGCTGCTTGATCTTCTCC‐3′ (reverse); HDAC2: 5′‐GCCAAGTCAGAACAACTCAGC‐3′ (forward) and 5′‐GTCCTCAAACAGGGAAGGTT‐3′ (reverse); HDAC3: 5′‐TTGAAGATGCTGAACCATGC‐3′ (forward) and 5′‐TGGCCTGCTGTAGTTCTCCT‐3′ (reverse). The analyses of relative mRNA expression were carried out using 2−ΔΔCt method 15.

### HDAC1 activity assay

The nuclear proteins were extracted as mentioned previously. HDAC1 activities of the nuclear protein extraction were determined using a HDAC1 Activity Fluorometric Assay kit (BioVision, Mountain View, CA, USA) according to the manufacturer's instructions. Test samples (30 μg of nuclear extract) were diluted to 85 μl (final volume) of ddH2O in each well (for background reading, only 85 μl ddH2O was added). The samples were read in a fluorescence plate reader (Ex/Em = 380/500 nm). The signal should be stable for several hours at room temperature. Histone deacetylase activities were expressed as the relative fluorescence units per μg protein sample. The HDAC1 activities were expressed as relative fluorescence units (RFU).

### Chromatin immunoprecipitation (ChIP) assay

After homogenization of cardiac tissues, formaldehyde (1%) (Sigma‐Aldrich, St. Louis, MO, USA) was added to the samples to cross‐link protein–DNA complexes. ChIP trials were conducted using a ChIP assay kit (Millipore, MA, USA). The DNAs were ultrasonically cut into small fragments (200–1000 bp). Then, the protein–DNA complexes were recruited and precipitated using monoclonal antibody against acetylated histone 3 lysing 9, HDAC1, HDAC2, HDAC3, GATA4 and Mef2c (Abcam10812, Abcam7028, Abcam12169, Abcam7030, Santa‐SC1237 and Abcam‐79436). Anti‐RNA polymerase was used as a positive control, and mouse IgG was used as a negative control. Input groups which contain the protein–DNA complexes without antibodies were collected to show the total DNA in the samples. And then, the crossing link of proteins and DNAs were removed, and the DNAs were extracted. Specific primers were designed to determine the acetylation level of H3K9, and binding levels of HDAC1, HDAC2, HDAC3, GATA4 and Mef2c in proximal promoter regions of cTnI for quantitative real‐time PCR (qPCR) analysis. The primer sequence was as follows: cTnI 5′‐CCAACTGGAGCTTTGCACACG‐3′ (forward) and 5′‐AGGACACTGAGATAAGGGGCG‐3′ (reverse).

### Statistical analysis

All data were expressed as mean ± S.D. and statistically analysed by one‐way anova. The differences were considered statistically significant when *P* < 0.05.

## Results

### EGCG improved heart functions of ageing mice

Cardiac functions were evaluated by high‐frequency ultrasound, noninvasively. As shown in Table 1, indicators of heart systolic function, such as EF value, FS value and left ventricular isovolumic contraction time (IVCT), did not show any significant changes between young adult mice and aged mice (*P* > 0.05). However, aged mice showed a significant prolongation of left ventricular isovolumetric relaxation time (IVRT) compared with young adult mice (12.91 ± 1.25 ms *versus* 15.33 ± 0.99 ms, *P* < 0.05). The E/A ratio measured with Doppler was significantly reduced in the 18‐month‐old mouse group (1.66 ± 0.99) compared to that in the 3‐month young mouse group (1.31 ± 0.07).

**Table 1:** Cardiac function evaluation

Parameters	18M	18M + DMSO	18M + EGCG	3M
Body weight (g)	23.59 ± 1.32	23.59 ± 1.32	22.83 ± 2.07	24.04 ± 1.47
Heart rate (beats/minute)	478 ± 12	489 ± 9	483 ± 11	479 ± 8
LV end‐diastolic				
IVS (mm)	0.91 ± 0.16	0.94 ± 0.16	0.85 ± 0.23	0.92 ± 0.20
LVID (mm)	3.01 ± 0.48	3.07 ± 0.41	3.23 ± 0.68	3.05 ± 0.43
LV PW (mm)	0.84 ± 0.28	0.85 ± 0.32	0.78 ± 0.14	0.78 ± 0.09
LV volume (µl)	36.12 ± 13.14	37.01 ± 10.92	44.46 ± 21.92	37.41 ± 10.39
LV end‐systolic				
IVS (mm)	1.72 ± 0.40	1.75 ± 0.46	1.53 ± 0.16	1.64 ± 0.23
LVID (mm)	1.26 ± 0.18	1.27 ± 0.15	1.49 ± 0.43	1.41 ± 0.22
LV PW (mm)	1.43 ± 0.33	1.48 ± 0.36	1.45 ± 0.27	1.34 ± 0.14
LV volume (μl)	3.93 ± 1.31	4.04 ± 1.11	4.71 ± 3.60	5.39 ± 1.99
Ejection fraction (%)	88.92 ± 2.44	88.93 ± 2.94	86.60 ± 3.40	85.49 ± 3.47
LV fractional shortening (%)	57.95 ± 3.52	58.15 ± 4.57	55.58 ± 5.23	53.56 ± 4.57
Stroke volume (µl)	32.18 ± 12.14	33.83 ± 11.25	37.60 ± 17.20	32.02 ± 9.10
LV mass corrected (mg)	68.87 ± 26.82	74.69 ± 30.47	57.86 ± 23.71	67.28 ± 18.10
Mitral pulse Doppler				
E/A	1.31 ± 0.07*	1.33 ± 0.06	1.60 ± 0.07	1.66 ± 0.09
IVRT (ms)	15.33 ± 0.99*	15.74 ± 1.09	13.21 ± 1.09	12.91 ± 1.25*
IVCT (ms)	8.01 ± 0.62	8.47 ± 0.77	8.43 ± 1.00	8.47 ± 1.33

Values are expressed as mean ± SE for each group. LV, left ventricle; IVS, intraventricular septum; LVID, left ventricular internal diameter; PW, posterior wall thickness of LV; IVRT, isovolumetric relaxation time; IVCT, isovolumetric contraction time; EGCG, epigallocatechin‐3‐gallate. Statistical significance was determined by anova followed by *post hoc* Student–Newman–Keuls (SNK) tests. *P < 0.05, compared with the 18M + EGCG and 3M groups.

Mice were treated with EGCG or DMSO (dissolvent control) for 8 weeks treatment at 16 month. After treatment, IVRT of 18‐month‐old mice (13.21 ± 1.09 ms) was significantly reduced compared with DMSO‐treated (15.74 ± 1.09 ms) and untreated mice (15.33 ± 0.99 ms). E/A ratio was increased significantly in the EGCG‐treated group (1.60 ± 0.07) compared to that in the DMSO‐treated group (1.33 ± 0.06) and untreated group (1.31 ± 0.07). IVRT is considered to be the most sensitive Doppler index to detect impaired relaxation 16, 17.

### EGCG may prevent sarcomere dissolution in cardiomyocytes

**Figure 1:**
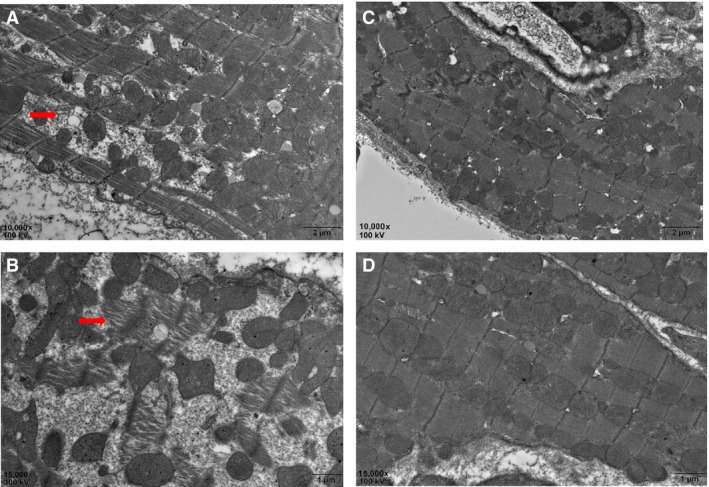
EGCG treatment prevents sarcomere dissolution in cardiomyocytes. Red arrow shows sarcomere dissolution in the hearts of 18‐month‐old mice (**A** 10,000×; **B** 15,000×); (**C** and **D**) the cardiac ultrastructure after EGCG treatment. Scale bars in A and C = 2 μm; B and D = 1 μm. EGCG: epigallocatechin‐3‐gallate.

We compared the ultrastructures of cardiac tissues of 18‐month‐old mice with or without EGCG treatment by transmission electron microscope. Figure 1A and B shows the ultrastructures of cardiomyocytes in 18‐month‐old mice; significant sarcomere dissolution was found as we reported previously (indicated by red arrows). Interestingly, after EGCG treatment, no obvious sarcomere dissolution was found (Fig. 1C and D).

**Figure 2:**
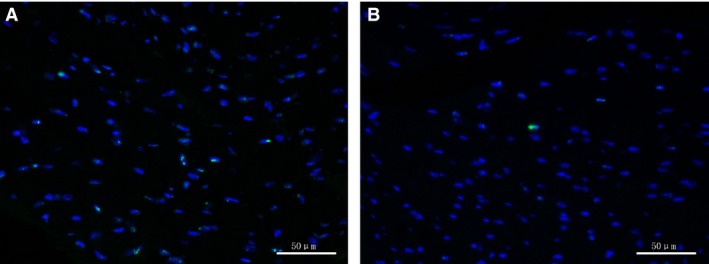
EGCG prevents myocardial apoptosis in ageing hearts. TUNEL assay results showed that EGCG treatment decreased apoptosis myocardial cells in ageing hearts. (**A**) 18M group; (**B**) 18M + EGCG group. Blue: DAPI; green: TUNEL. Scale bars, 50 μm. EGCG: epigallocatechin‐3‐gallate.

EGCG prevents myocardial apoptosis in ageing hearts. The heart will undergo an increase in apoptosis during ageing 18. So we used TUNEL assay to evaluate myocardial apoptosis levels in various groups. Figure 2 shows that apoptosis cells increased in ageing hearts, and after EGCG treatment, positively stained cells in the 18M + EGCG group were significantly higher than those in the 18M group.

### EGCG treatment increased cTnI expression in ageing hearts

**Figure 3:**
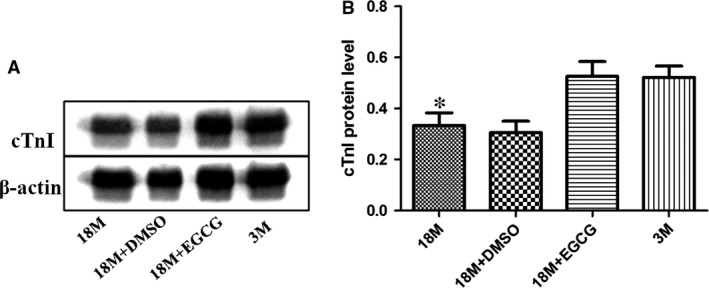
EGCG treatment increases cTnI protein levels in ageing hearts. **(A)** Western blotting analysis using cTnI and cTnT antibodies. β‐Actin was used as protein loading control. **(B)** A summary of Western blotting results of cTnI. Values are expressed as mean ± S.D. from four separate experiments. Statistical significance was determined by anova followed by least significant difference (LSD) tests. *P < 0.05 as compared with the 18M + EGCG and 3M groups. EGCG: epigallocatechin‐3‐gallate.

It is known that cTnI is closely related to cardiac diastolic function. As shown in our most recent study, cTnI decreased significantly in ageing hearts; meanwhile, the previous results showed that EGCG could improve cardiac functions of ageing mice. So the contents of cTnI were tested by Western blotting. As illustrated in Figure 3, cTnI protein level decreased significantly in the hearts of the 18‐month group compared to that in the 3‐month group; interestingly, it increased notably after EGCG intervention.

**Figure 4:**
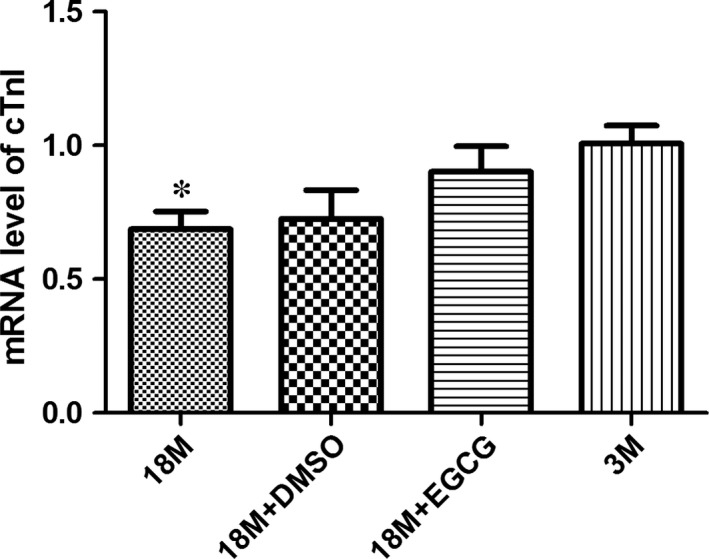
EGCG treatment increases cTnI mRNA levels in ageing hearts. The expression pattern of cTnI mRNA in the hearts of mice at various ages and before and after EGCG intervention. The results are expressed as mean ± S.D. from at least three separate experiments. Statistical significance was determined by anova followed by least significant difference (LSD) tests. *P < 0.05 as compared with the 18M + EGCG and 3M groups. EGCG: epigallocatechin‐3‐gallate.

We then used qPCR assay to detect the mRNA level of cTnI. As shown in Figure 4, similar to its protein expression pattern, cTnI mRNA level was reduced in aged period and up‐regulated by EGCG treatment.

### Determination of HDACs enzyme expression

**Figure 5:**
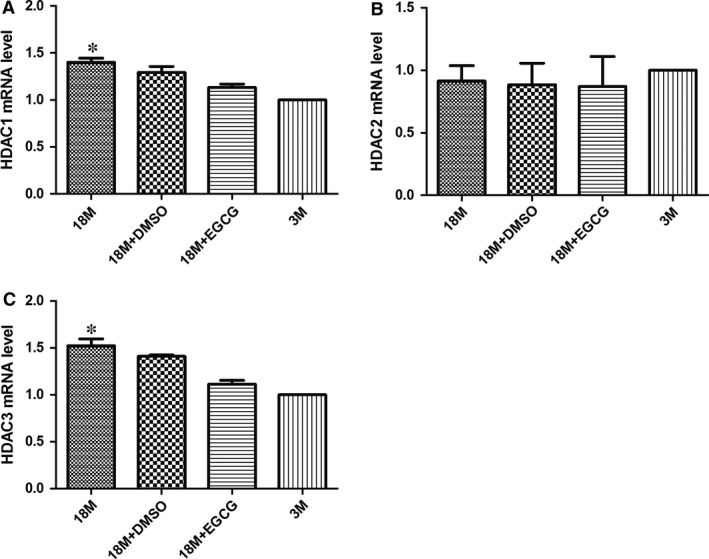
EGCG inhibits class I HDACs mRNA levels in ageing hearts. mRNA levels of HDAC1 (**A**), HDAC2 (**B**) and HDAC3 (**C**) were detected in the four groups. The values of the 3M group are standardized as 1. Values are expressed as mean ± S.D. from three separate experiments. Statistical significance was determined by anova followed by least significant difference (LSD) tests. *P < 0.05 as compared with the 18M + EGCG and 3M groups. EGCG: epigallocatechin‐3‐gallate.

Recently, studies showed that EGCG could inhibit class I HDAC expression and/or activities *in vitro* 10, 12; meanwhile, it could increase acetylated histone 3 lysine 9 levels and enhance p53 gene transcriptional activity 11, 12, 19. Therefore, we detected class I HDAC expression levels by the qPCR method. As illustrated in Figure 5, mRNA levels of HDAC1 (A) and HDAC3 (C) went up in ageing hearts, and EGCG inhibited HDAC1 and HDAC3 expression after treatment. However, HDAC2 showed no significant changes among these groups (B).

### Determination of histone acetylation level in the key cis‐element of cTnI gene's promoter

**Figure 6:**
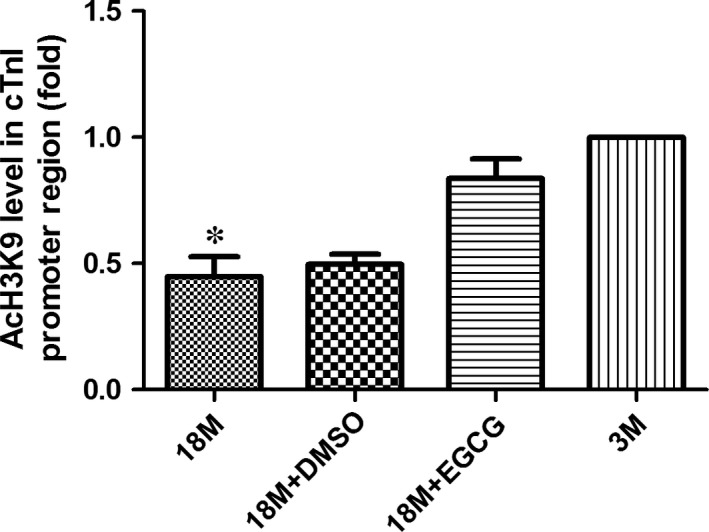
EGCG reverses the hypoacetylation of AcH3K9 near the proximal promoter of cTnI in ageing hearts. Acetylation levels of specific lysine 9 amino acid in H3 (AcH3K9) are detected near the promoter of cTnI. The values of the 3M group are standardized as 1. Values are expressed as mean ± S.D. from three separate experiments. Statistical significance was determined by anova followed by least significant difference (LSD) tests. *P < 0.05 as compared with the 18M + EGCG and 3M groups. EGCG: epigallocatechin‐3‐gallate.

As a crucial part of epigenetic regulation, modification of histone acetylation/deacetylation is a reversible dynamic process. Histone acetylation modification can weaken the electrostatic attraction between DNA and histone, reducing chromatin compaction, and then increase gene transcription activity 14, 20. Our previous study found that histone acetylation events, especially hypoacetylation of histone 3 lysine 9 near the proximal promoter of cTnI, played a critical role in cTnI down‐regulation of ageing heats 8. Therefore, in the present study, ChIP‐qPCR assays were employed to detect AcH3K9 levels near the key cis‐element of cTnI gene's proximal promoter. As shown in Figure 6, AcH3K9 levels near the promoter of cTnI were lower in 18‐month‐old mice compared with 3‐month‐old mice. Hypoacetylation levels of cTnI's promoter were reversed by EGCG. However, there were no significant differences of total acetylated histone 3 in various groups (Fig. S1).

### Determination of HDAC binding levels in the key cis‐element of cTnI gene's promoter

**Figure 7:**
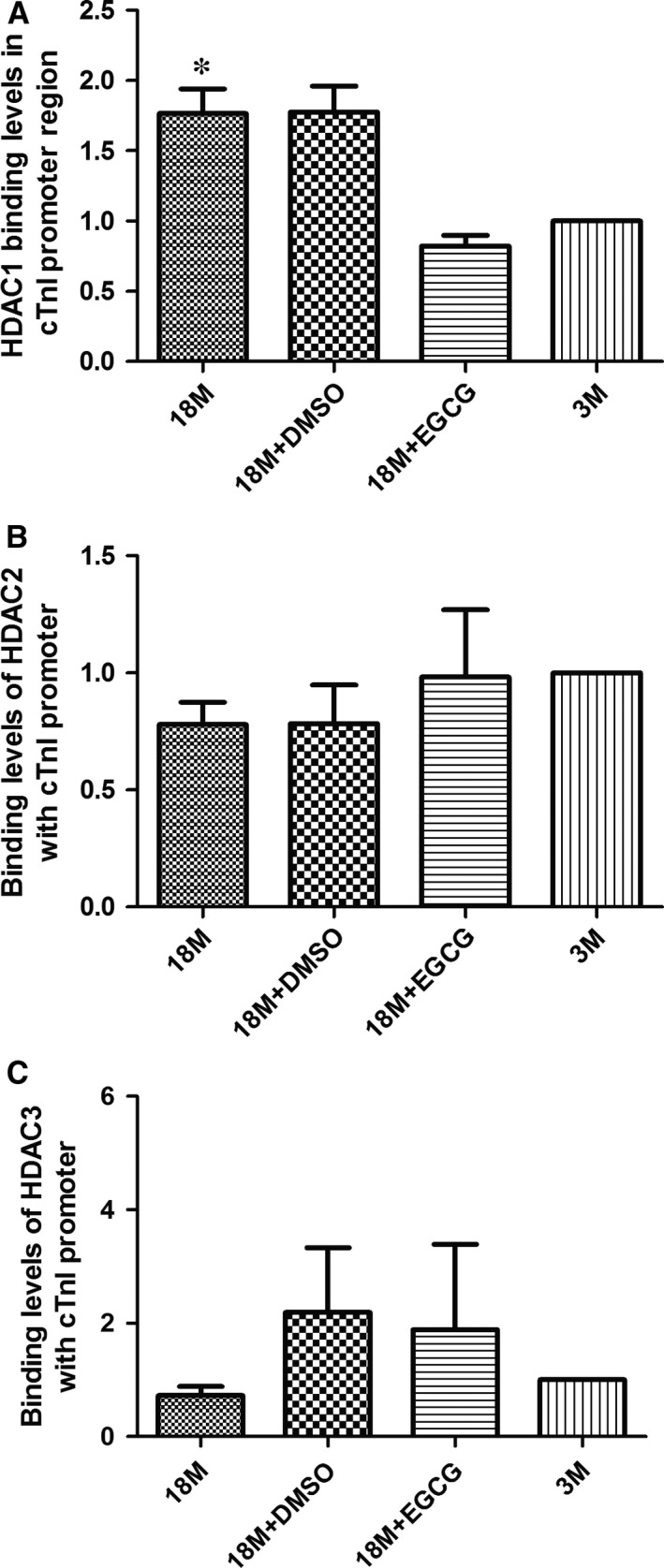
EGCG inhibits HDAC1 binding levels with cTnI's proximal promoter. Binding levels of HDAC1 (**A**), HDAC2 (**B**) and HDAC3 (**C**) with the cTnI's promoter region were detected in the four groups. The values of the 3M group are standardized as 1. Values are expressed as mean ± S.D. from three separate experiments. Statistical significance was determined by anova followed by least significant difference (LSD) tests. *P < 0.05 as compared with the 18M + EGCG and 3M groups. EGCG: epigallocatechin‐3‐gallate.

Histone acetylation is a dynamic process and is mediated by histone acetylase (HAT) and HDAC. ChIP‐qPCR assays were used to determine the binding levels of HDAC1, HDAC2 and HDAC3 with cTnI's proximal promoter. Figure 7A shows the binding levels of HDAC1 with cTnI's promoter. The binding levels of HDAC1 were decreased in ageing hearts, which may correlate with down‐regulation of AcH3K9. Surprisingly, HDAC1 binding levels in cTnI's promoter were raised by EGCG treatment. However, the binding levels of HDAC2 (B) and HDAC3 (C) showed no significant differences among each group.

### Determination of HDAC1 activity

**Figure 8:**
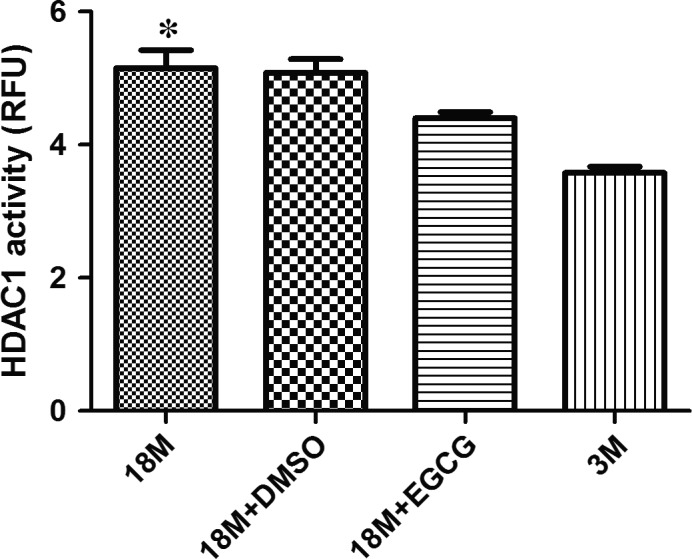
HDAC1 activity analysis before and after EGCG treatment. The expression pattern of HDAC1 activity in the hearts of mice at various ages and before and after EGCG intervention. The results are expressed as mean ± S.D. from at least five separate experiments. Statistical significance was determined by anova followed by least significant difference (LSD) tests. *P < 0.05 as compared with the 18M + EGCG and 3M groups. EGCG: epigallocatechin‐3‐gallate.

EGCG could inhibit HDAC1 expression and also reduce its binding levels with cTnI's proximal promoter. Next, we determined its activity in each group. Figure 8 shows HDAC1 activity in various groups. EGCG depressed HDAC1 activity; however, there were still significant differences between the 3M and 18M + EGCG group.

### Binding levels of transcription factors in proximal promoter of cTnI gene

**Figure 9:**
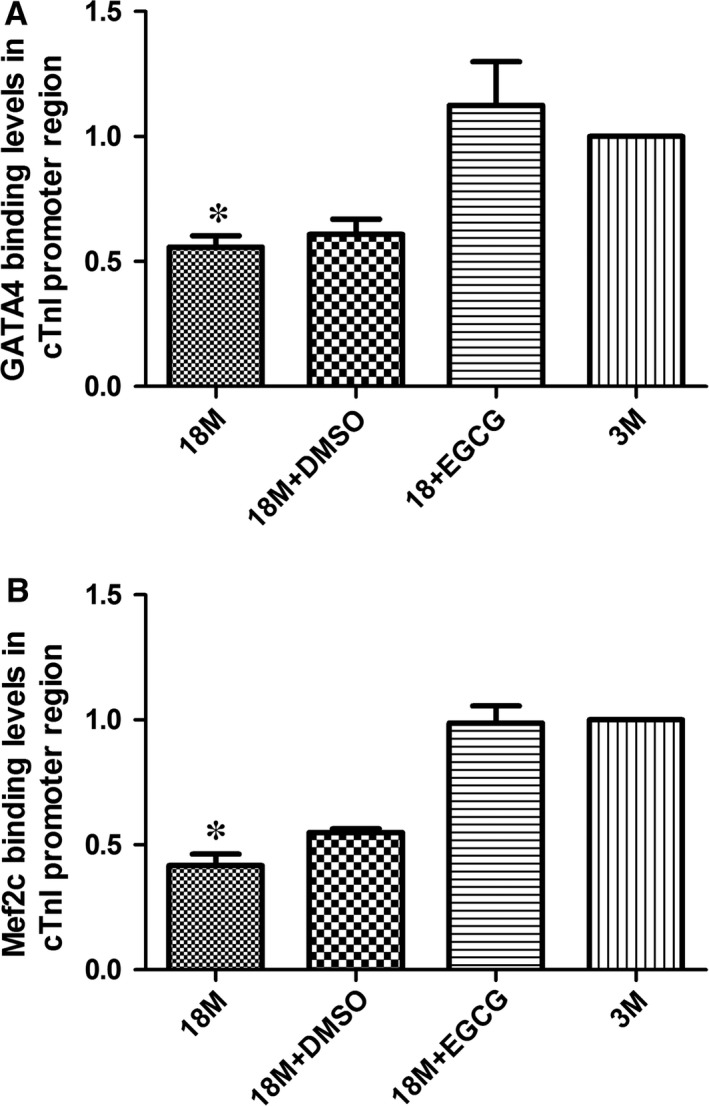
EGCG up‐regulates the binding of GATA4 and Mef2c with the proximal promoter regions of cTnI. Transcription factor GATA4 binding with GATA elements of cTnI's promoter is measured in the cardiac samples in mice (**A**). Transcription factor Mef2c binding with Mef2 element in the promoter of cTnI is measured in cardiac samples in mice (**B**). Values are expressed as mean ± S.D. from three separate experiments. Statistical significance was determined by anova followed by least significant difference (LSD) tests. *P < 0.05 as compared with the 18M + EGCG and 3M groups. EGCG: epigallocatechin‐3‐gallate.

Two kinds of cis‐elements of cTnI's proximal promoter, A/T‐rich and GATA elements, were demonstrated to play considerable roles in the regulation of cTnI gene transcription 21, 22. Mutation of each of these elements markedly reduces gene activation 23. Cardiac core transcription factors, Mef2c and GATA4, specifically bind to these elements, respectively. So, ChIP assays were also used to determine the binding levels of transcription factors with the proximal promoter of cTnI. As displayed in Figure 9, binding levels of GATA4 (A) and Mef2c (B) with proximal promoter of cTnI were both reduced in ageing hearts and may be caused by hypoacetylation of histones. Due to the reverse effect of EGCG on acetylation modification in cTnI gene's histones, binding levels of GATA4 and Mef2c were increased significantly after EGCG treatment.

## Discussion

Heart failure is typically classified as either systolic, in which there is reduced ventricular pump function, or diastolic, which is characterized by impaired cardiac relaxation and abnormal ventricular filling. Cardiac ageing is commonly associated with diastolic heart failure, which is also referred to as heart failure with preserved ejection fraction (HFpEF)^24^. Approximately half of the 5 million heart failure patients in the United States have been diagnosed with HFpEF, and more than 90% of HFpEF patients are over the age of 60 at the time of diagnosis 25, 26. Large clinical trials using standard‐of‐care systolic heart failure medications have failed to demonstrate efficacy in patients with HFpEF 27, 28, highlighting the need to better understand the molecular basis of HFpEF so that novel therapeutic interventions can be developed.

At least one study has reported that the content of cTnI in left ventricular myocardial cells decreased in older men with or without cardiac disease 29. The deficiency of cTnI in the heart can result in diastolic dysfunction and sudden death in cTnI knockout mice 30. Further studies have revealed that cTnI is critical in the regulation of cardiac function, especially diastolic function. Loss of cTnI or mutations on cTnI has been confirmed to be associated with impaired relaxation and diastolic heart failure 7, 31. Our recently published data showed that cTnI decrease in ageing hearts might be one of the reasons that caused diastolic dysfunction of aged mice 8. Moreover, we found that acetylation of histone near the promoter region of cTnI gene played an important role in the regulation of cTnI expression in the heart at different ages. However, the cause of hypoacetylation of H3K9 near cTnI's promoter in ageing hearts is still less obvious. In the present study, we found that HDAC1 and HDAC3 were increased in ageing hearts, and also the binding levels of HDAC1 with cTnI's promoter went up significantly. These data indicated that HDAC1 might be involved in the regulation of AcH3K9 hypoacetylation near cTnI's promoter. HDACs can enhance the electrostatic attraction between DNA and histone, increasing chromatin compaction, and then may decrease the binding affinity of transcription factors with genes' promoter 14, 23.

Histone acetylation is one of the most important forms that may cause chromatin remodelling, and the hypoacetylation of H3K9 near cTnI's proximal promoter makes the chromatin compaction and may affect the binding affinities of transcription factors with the key cis‐elements in the proximal promoter. Our results showed that the binding affinities of GATA4 and Mef2c with cTnI's promoter were reduced significantly in ageing hearts, and it was reversed by EGCG treatment through up‐regulating AcH3K9. EGCG induced an increase of cTnI may explain that it could improve cardiac diastolic function in the ageing hearts, a significant decrease of IVRT and an ascent of E/A ratio.

Emerging data have revealed numerous mechanisms by which HDAC inhibitors benefit the heart, including suppression of oxidative stress and inflammation, inhibition of MAP kinase signalling, and autophagic flux 32. Class I HDACs could repress antihypertrophic genes, such as Inpp5f and KLF4 33, and have been found overexpressed in hypertrophic cardiomyopathy 34. Meanwhile, histone acetylation has been considered as a hallmark of ageing 35. In our study, we found that the expression levels HDAC1 and HDAC3 were increased in the hearts of 18‐month‐old mice, suggesting that HDACs may also involve in cardiac ageing and might be considered as markers of cardiac ageing. In the present study, we found that EGCG could inhibit HDAC1 expression and its activity and also reduce its binding levels with cTnI's proximal promoter and that it might cause an increase in AcH3K9 near the proximal promoter of cTnI, and then, we observed that the binding levels of GATA4 and Mef2c with cTnI's key cis‐elements were increased, the expression of cTnI was enhanced and cardiac function was improved.

On the other side, we determined p66Shc expression levels and heart cell apoptosis levels before and after EGCG treatment. P66Shc is considered to be one of the most classic markers of cardiac ageing 36, and also it is strongly related to myocardial oxidative stress 37. Interestingly, p66Shc decreased significantly after EGCG intervention (Fig. S2). We also found that EGCG inhibited cell death in ageing mice. However, the underlying mechanisms are still unknown. EGCG is also known as an antioxidant and some studies showed its anti‐ageing effect 38, 39, and it may partly explain the decrease in p66Shc and cell apoptosis. It is known that fibrosis and cardiac hypertrophy are strongly associated with heart diastolic dysfunction, because both of them can limit the diastolic function due to an increased myocardial stiffness. In our study, we also assessed cardiac fibrosis level before and after EGCG treatment, collagen production was increased in ageing hearts, and EGCG treatment, to some extent, decreased cardiac fibrosis (Fig. S3A–D). However, we did not find cardiac hypertrophy in ageing hearts (Fig. S3E–F).

Dietary polyphenols from green tea and its major constituent, EGCG, have been demonstrated to possess therapeutic activity in human cancer and a number of other diseases 40. And now EGCG has been approved as a healthy product for sale. In conclusion, our data indicate that EGCG might be a new therapy method for age‐related cardiac diastolic dysfunction.

## Funding source

This study was supported by research grants from Natural Science Foundation of China (Grant Number: 81670212; 81270234).

## Conflict of interest

The authors have no conflict of interests to declare.

## Supporting information


**Fig. S1** Acetylated histone3 levels in various groups.

**Fig. S2** EGCG treatment increased cTnI mRNA levels in aging hearts.

**Fig. S3** Morphological and histological examination of the heart.
