# Supplementary material for: Epigallocatechin gallate reverses cTnI‐low expression‐induced age‐related heart diastolic dysfunction through histone acetylation modification
Source: J Cell Mol Med. 2017 Apr 6;21(10):2481–90. doi: 10.1111/jcmm.13169 (PMC5618683; doi:10.1111/jcmm.13169)

**Figure S1 Acetylated histone3 levels in various groups**

(A)Western blotting analysis using acetylated histone3 (AcH3) antibodies. Total hisone3 was used as protein loading control. Summary of Western blotting results of AcH3 are shown in Figure S1B. Values are expressed as means ± SD from 3 separate experiments. Statistical significance was determined by ANOVA followed by Least—Significant Difference (LSD) tests. No significant differences of AcH3 in various groups.


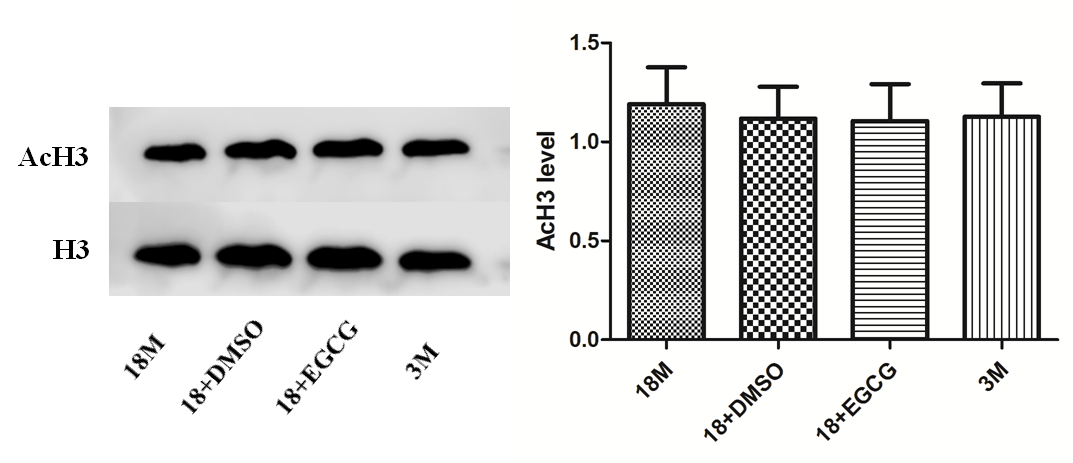

Supplement: Supplementary file 1 — Fig. S1 Acetylated histone3 levels in various groups. [file JCMM-21-2481-s001.docx]
