# Supplementary material for: Epigallocatechin gallate reverses cTnI‐low expression‐induced age‐related heart diastolic dysfunction through histone acetylation modification
Source: J Cell Mol Med. 2017 Apr 6;21(10):2481–90. doi: 10.1111/jcmm.13169 (PMC5618683; doi:10.1111/jcmm.13169)

**Methods**

p66Shc mRNA expression levels in each group

The real-time PCR was carried out to measure p66Shc mRNA levels in each group. β-actin was used as the endogenous “house-keeping” gene to normalize the RNA sample levels. The primer sequences of cardiac-specific genes and controls were designed as follows: p66Shc: 5’- AAGGTGTGGTTCGGACAAAG-3’ (forward) and 5’- GGGTTGCTGTAGGCACAGTT-3’ (reverse).

**Result**

EGCG treatment decreased p66Shc expression in aging hearts

It is known that p66Shc is one of the specific myocardial aging markers. And also, it is closely related to oxidative stress in hearts. So we determined p66Shc expression levels in each group. As illustrated in Figure S2, p66Shc was increased significantly in aging hearts (18m) compared with 3m, and EGCG treatment may decrease its expression.

**Figure S2 EGCG treatment increased cTnI mRNA levels in aging hearts**

Figure S2 displays the expression pattern of p66Shc mRNA in the hearts of mice at various ages and before and after EGCG intervention. The results are expressed as mean ± SD from 6 separate experiments. Statistical significance was determined by ANOVA followed by Least—Significant Difference (LSD) tests. *p<0.05 as compared with with 18M+EGCG and 3M groups.


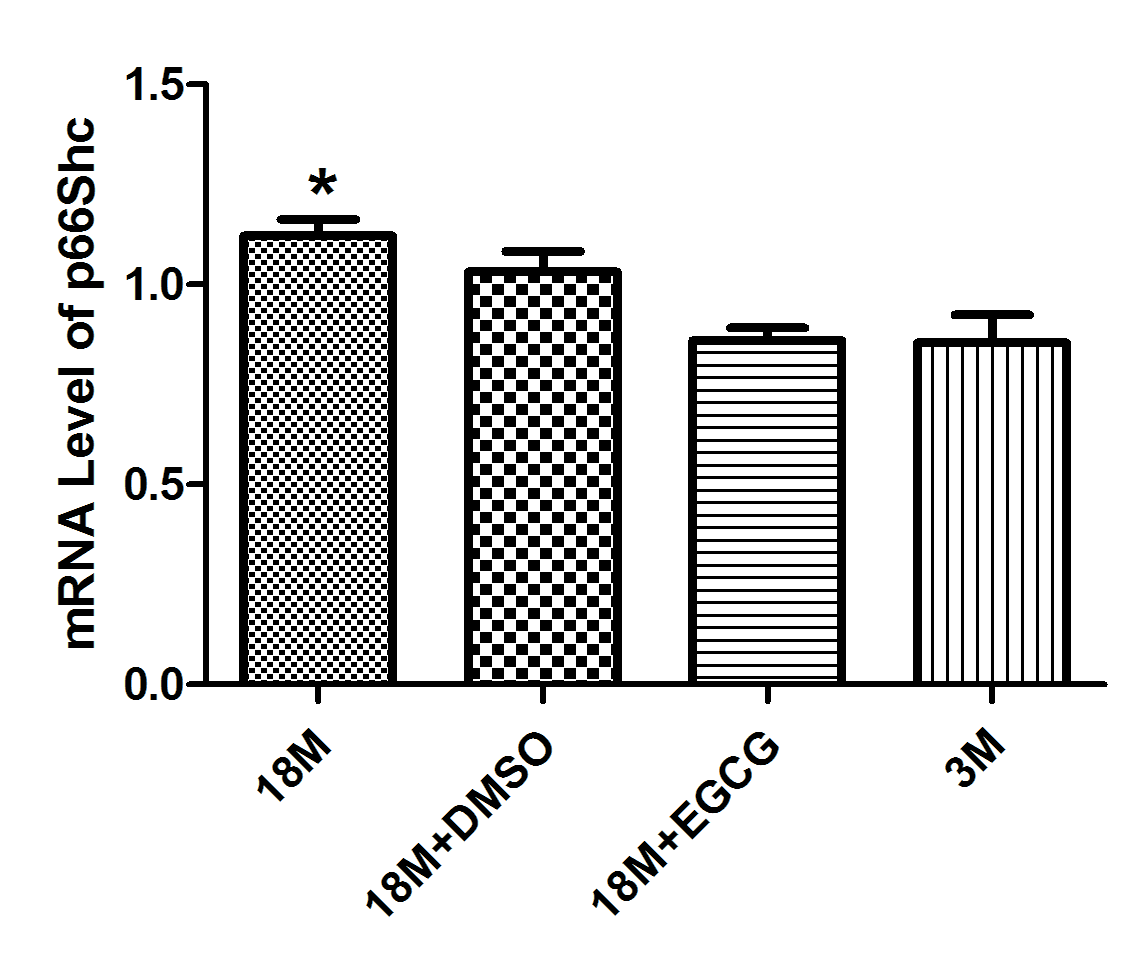

Supplement: Supplementary file 2 — Fig. S2 EGCG treatment increased cTnI mRNA levels in aging hearts. [file JCMM-21-2481-s002.docx]
