# Supplementary material for: Epigallocatechin gallate reverses cTnI‐low expression‐induced age‐related heart diastolic dysfunction through histone acetylation modification
Source: J Cell Mol Med. 2017 Apr 6;21(10):2481–90. doi: 10.1111/jcmm.13169 (PMC5618683; doi:10.1111/jcmm.13169)
Supplement: Supplementary file 3 — Fig. S3 Morphological and histological examination of the heart. [file JCMM-21-2481-s003.docx]

**Method**

The heart tissues of each group were collected and fixed in 4% paraformaldehyde at least 24 hours. After dehydration of tissue samples, paraffins were used for tissue-embedding. Section through the hearts were cut by machine (Leica, Solms, German). And then the tissue samples section were stained as following the H&E staining steps, and photographed by the microscope (Nikon, Tokyo, Japan). Masson staining was also performed to detect fibrosis in the heart sections.

**Result**

Morphological evaluation

It is known both cardiac fibrosis and hypertrophy are closely related to diastolic dysfunction. We then observed fibrosis and hypertrophy levels among each group. Consistent with others results, cardiac fibrosis were found in aging hearts, and EGCG treatment decrease cardiac fibrosis in some extent (Figure S3 A-D). However, we did not found cardiac hypertrophy in aging hearts (Figure S3 E-F)

.

**Figure S3 Morphological and histological examination of the heart**

Figure S3 showed fibrosis levels (blue staining of collagen) in 18M (A), 18M+D (B), 18M+E (C) and 3M (D) hearts, EGCG treatment decrease cardiac fibrosis in some extent. H&E staining of cardiac sections were shown in picture E and F, no obvious cardiac hypertrophy was found between 18M (E) and 3M (F) hearts. Scale bars= 100μm.

**
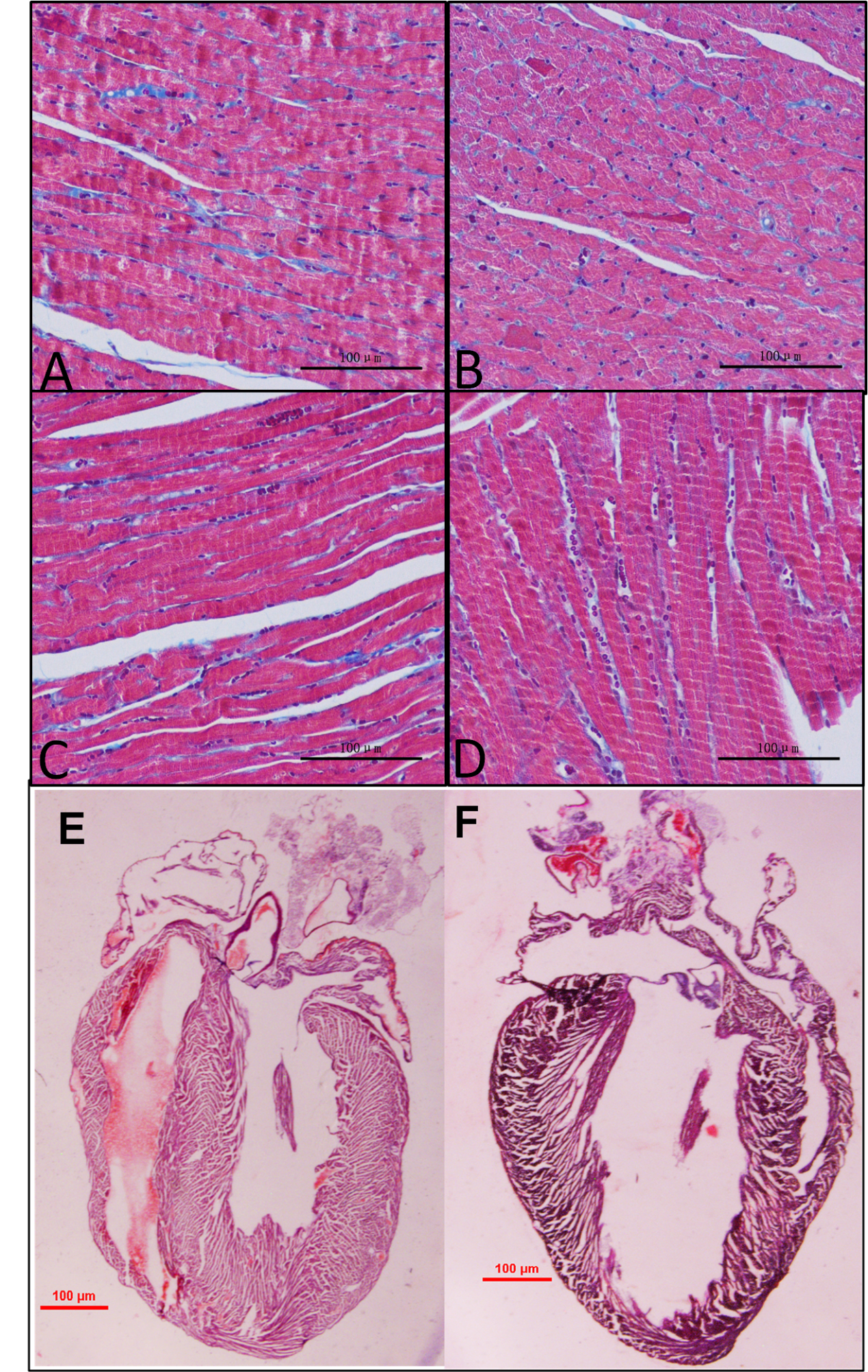
**
